# A Mixed‐Reality Navigation Simulator for Training Infiltration Procedures of the Spine: Evaluation in Kambin Triangle Location

**DOI:** 10.1002/rcs.70139

**Published:** 2026-02-13

**Authors:** César F. Domínguez‐Velasco, Jessica Alatorre‐Flores, Jorge A. Pérez‐Terrazas, Efrain Albor‐Ramírez, Felipe Camarillo‐Juárez, Marino A. Capurso‐García, Fabrizio Cutolo, Vincenzo Ferrari, Miguel A. Padilla‐Castañeda

**Affiliations:** ^1^ Applied Sciences and Technology Institute ICAT, National Autonomous University of Mexico UNAM, Ciudad Universitaria Mexico Mexico; ^2^ Orthopedics Service Unit General Hospital of Mexico ‘Dr. Eduardo Liceaga’ Mexico Mexico; ^3^ Orthopedics Service Unit General Hospital of Tijuana Tijuana Baja California Mexico; ^4^ Directorate of Education General Hospital of Mexico ‘Dr. Eduardo Liceaga’ Mexico Mexico; ^5^ EndoCAS Interdipartimental Center University of Pisa Pisa Italy; ^6^ Department of Information Engineering University of Pisa Pisa Italy

**Keywords:** augmented reality, fluoroscopy, mixed‐reality, neurosurgery, orthopedics, spine, surgery navigation, surgery simulation

## Abstract

**Background:**

During minimally invasive surgeries, the Kambin Triangle infiltration procedure is primarily used as a safe anatomical access route to critical spine structures. Inadequate location of final infiltrations can be critical for inexperienced residents.

**Methods:**

A mixed reality (MR) navigator simulator for spine infiltration was developed. It combines virtual fluoroscopy simulation (FS) and augmented reality views with 3D‐printed lumbar spine patient models. Ten residents of orthopaedics tested the system.

**Results:**

Residents showed more infiltration success under MR than FS but with higher risky contacts at the nerve roots. Residents reported an improved understanding of the anatomy and clinical case during MRI guidance and greater ease in locating target points in this condition.

**Conclusion:**

The results demonstrate the advantages of combining MR with FS for training minimally invasive spinal procedures to progressively train in a scenario close to reality once the anatomical and safety aspects of the approach have been understood.

## 1 Introduction

**FIGURE 1:**
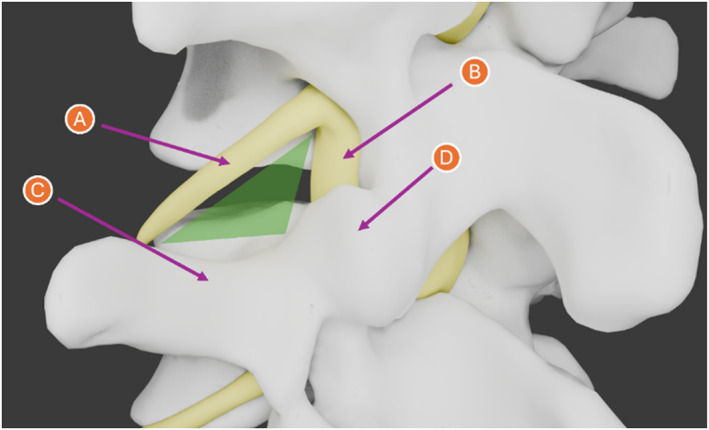
Schematic representation of the lumbar spine anatomy. The diagram highlights the anatomical boundaries relevant to the procedure: (A) Exiting nerve root. (B) Traversing nerve root. (C) Transverse process, and (D) Superior articular process. The Kambin's Triangle (KT), identified in the literature as a safe access zone, is highlighted in green.

Identification of the posterolateral corner of the disc is essential for performing minimally invasive surgeries for the treatment of low back pain caused by herniated discs in the spine [1], such as percutaneous transforaminal endoscopic lumbar interbody fusion and other lumbar spine surgeries. The posterolateral corner of the disc or Kambin triangle (KT) serves as a safe region to access the intervertebral discs (Figure 1).

Accurate identification and localisation of delicate structures within this region are essential to maintain the safety and efficacy of surgical procedures and infiltration. Short operating times, less blood loss, and improved early patient recovery have been reported as advantages. However, there is a reported risk of postoperative motor weakness due to nerve root injury caused by cannula placement in the KT zone close to the nerve root [2, 3]. Other risks include vascular injury [4, 5] and inadvertent intradiscal injection [4]. In addition, it is important to consider that the KT's anatomical variations in shape and size may pose challenges [6]. Therefore, careful preoperative planning and imaging studies are necessary to avoid complications [7].

Available methods for accessing KT include imaging patient studies, such as computed tomography (CT) and magnetic resonance imaging (MRI), which are used to define the surgical corridor and guide instrument placement [8]. Fusion of the two imaging modalities has been reported to improve navigation accuracy and reduce adverse effects from injury. Intraoperative navigation is also used, combined with advanced imaging techniques such as nerve root segmentation and image fusion, to display real‐time bony and neural anatomy, which helps establish safe navigation within the CT [7]. Likewise, a preoperative MRI helps determine safe angles and entry points for transforaminal procedures, ensuring accurate definition of the intervertebral disk space [8].

Inadequate infiltration locations can be critical for inexperienced residents. Thus, exhaustive training in image‐guided techniques, such as fluoroscopy and intraoperative navigation, is strongly recommended for spinal infiltration interventions [9], allowing residents to acquire the skills for precise needle placement and minimal risk of complications [10].

Augmented reality (AR) has gained interest in recent years as a method for teaching specialised percutaneous and infiltration procedures. Some reported systems provide visuospatial assistance in catheter and needle placement tasks in high‐risk CT‐guided needle placement [11], ultrasound‐guided procedures [12], ventriculostomies in neurosurgery [13], interventional radiology [14, 15], trigeminal infiltration in pain management [16], thoracic drainage for pneumothorax treatment [17], and orthopaedic procedures of facet joint injections [18], among some clinical applications. Some incorporate commercial vision‐see‐through (VST) augmented reality devices, such as Microsoft's Hololens, which provide 3D visual information superimposed on physical models of patients, in a first‐person perspective, with the user directly viewing the surgical field in the model [19]. Another approach has been to provide augmented information on a screen, showing lateral views with 3D information superimposed on vision planes of the surgical scene. Although it does not allow to be focused staring directly at the patient model, it offers a simple setup that does not interfere with the field of view of the entire surgical scene and, in any case, has proven its usefulness [1, 13].

Regarding interventions on the spine, studies in the literature have integrated augmented reality, primarily for the visualisation of vertebrae and the placement of transpedicular screws [20, 21]. In contrast, few studies have addressed the recognition of critical anatomical structures due to the risk of contact, such as in [7], where the identification of Kambin's triangle and the nerve root was reported by merging MRI and angio‐CT studies. In a previous study [22], a case report of transfacet minimally invasive transforaminal lumbar interbody fusion using AR guidance was presented. From an educational perspective, it is essential to teach the infiltration technique, focussing on minimally invasive surgery, to improve the safety and efficacy of procedures in this anatomical region. This motivates the study of Kambin's triangle infiltration using AR and its relationship with fluoroscopy through simulation, as well as the identification of correct infiltrations and the possible injuries that can result from contact with the nerve root.

In this work, we present a study evaluating the experience of a group of residents using a mixed‐reality simulator for spinal infiltration procedures through Kambin's Triangle, combining a virtual‐reality fluoroscopy simulation and a mixed‐reality navigation scenario, with augmented and virtual reality views of the model. The overall opinion of residents over the system was positive, valuable, and helpful as a training tool. Residents expressed greater ease in understanding and executing the infiltrations with the help of mixed reality. Interestingly, residents had a higher rate of successful infiltrations during mixed‐reality navigation and more risky contacts at the nerve root than during fluoroscopy. This suggests the potential advantages of practicing under mixed‐reality guidance to better understand the anatomical aspects of the approach, followed by practice with simulated fluoroscopy, leading to progressively closer‐to‐reality training. However, these results emphasise the need to incorporate more effective visual guidance mechanisms that better warn residents of potential errors and risky manoeuvres during simulation before their first contact with real patients.

## 2 Materials and Methods

### 2.1 The Vertebrae Model

The clinical case of a patient was obtained from CT images acquired with a SOMATOM Definition Flash scanner (Siemens, Erlangen, Germany). The acquisition protocol included a slice thickness of 0.75 mm, a pixel spacing of 0.81 × 0.81 mm, a tube voltage of 80 kVp, and an exposure of 214 mAs. A 3D model based on meshes of the vertebrae was generated using the Segment Editor module in 3D Slicer [23]. Initial segmentation was performed using thresholding, followed by application of the Median and Gaussian smoothing filters to refine segmentation boundaries and eliminate acquisition artefacts. Additionally, a connected component analysis (Island selection) was utilised to preserve the desired topology and isolate the target vertebral structures from irrelevant floating artefacts.

Subsequently, the segmented meshes were manually refined using the open‐source Blender software [24]. This post‐processing stage involved mesh decimation, reducing the polygon count to 15% of the original density, and correcting non‐manifold geometry to ensure a topologically sound surface while preserving the anatomical fidelity of the lumbar spine. Then, a linked model of the entire lumbar region (L1–L5) was generated and considered a rigid body during the simulation.

**FIGURE 2:**
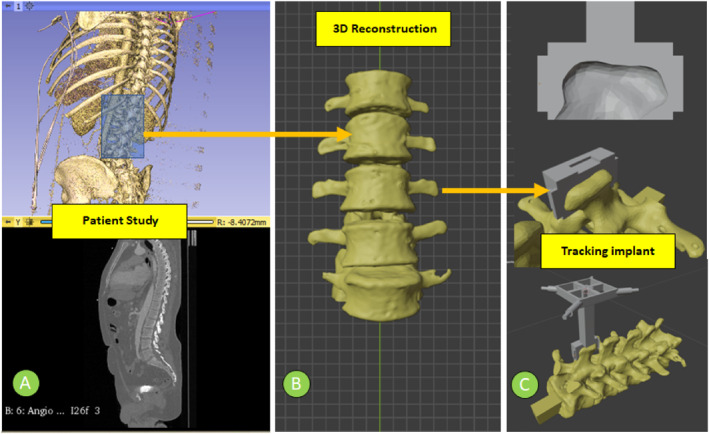
The construction process of the patient model. (A) Patient CT dataset segmentation and visualisation. (B) Three‐dimensional reconstruction of the lumbar spine. (C) Tracking implant design anatomically coupled to the spinal apophysis of the L1 Lumbar vertebra.

Additionally, to facilitate locating the spine model during navigation, an external implant was designed to be fixed to the spinous apophyses of the vertebrae. The implant incorporates a set of reflective spheres on its terminals to enable the optical tracking system to estimate the pose of the vertebrae in the virtual environment. The spine model and implant were manufactured in PLA using additive manufacturing via 3D printing on the Original Prusa i3 MK3S printer. High‐detail setting was used with a layer thickness of 0.15 mm. Additionally, a fill density of 20% was used to maintain the minimum stiffness required for the interaction. Maintaining the morphology of the anatomical models, the tool, and the casings as object markers allows the creation of navigation and simulation processes as performed in surgical procedures. Figure 2 illustrates the construction process of the lumbar spine model.

### 2.2 The Mixed‐Reality Navigator

**FIGURE 3:**
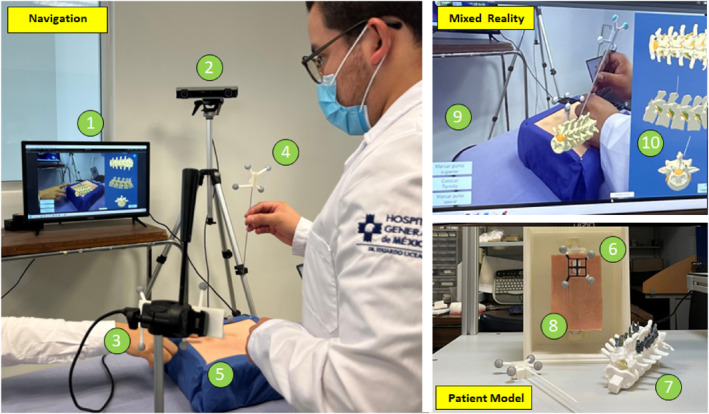
The complete setup of the mixed‐reality navigator for spine percutaneous minimally invasive procedures. (Left) The navigation scheme with its elements: (1) the mixed‐reality visualisation screen; (2) the optical tracker for monitoring in real‐time the pose of the elements of the navigator; (3) the RGB stereovision camera for the acquisition of the video frames of the real scene of the physical patient model; (4) a needle with a cross‐shape marker frame for tracking its pose in space; (5) the physical model of the patient. (Right Bottom) The elements of the patient model: (6) the tracking implant; (7) the 3D printed lumbar spine; (8) the silicon element mimicking the skin and the inner soft tissue. (Right Top) The elements of the mixed‐reality visualisation: (9) the augmented reality scene; (10) the virtual reality views of the spine model.

The system (Figure 3), named SpineNav, consists of the following modules: a mixed‐reality visualisation and navigation software; a physical model of the patient, including the printed spine model; a commercial optical tracking system (OTS) (Optitrack V120: Duo, Optitrack) for monitoring in real‐time the pose of the elements of the navigator; an RGB stereovision camera (Zed Mini, Stereolabs) for the acquisition of the video frames of the real scene of the physical patient model; and a rigid sterilisable aluminium needle. The needle and the stereo camera incorporate a rigid housing and a rigid reference frame with reflective inserts, respectively, to estimate the pose of both elements during navigation. Both frames were manufactured by 3D printing with the same specifications as the column model.

The patient model was built as a 3D‐printed compartment simulating the patient's lower dorsal torso, with the physical spine model immersed in polyurethane and an external soft silicone layer mimicking the skin.

To establish the stereo camera's location relative to the system origin, the camera's intrinsic and extrinsic parameters were estimated using a hand‐eye calibration. This resulted in the camera's position relative to its optical centre and in the correction of its optical distortion, generating a corrected and aligned augmented reality (AR) view during visualisation. The registration of the spine with respect to the same system origin was carried out by estimating the pose of the implant attached at one of the apophyses of the vertebrae, by means of the four reflective spheres on the implant head.

A procedure was performed to estimate the system's localisation errors. This involved a manual palpation procedure of a series of predefined reference landmark points, guided visually by virtual models projected onto the physical models in the augmented reality scene.

**FIGURE 4:**
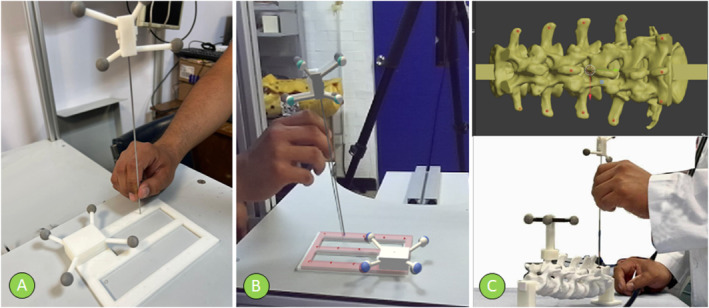
The calibration process of the system's error measurement consists of a manual probing of fiduciary points. The error is calculated as the root of the RMS distance between the known positions a set of fiducial points and the needle's tracked tip position. (A) Calibrating the tracked position error of the needle tip (*localisation error*) by probing known points over a 3D‐printed calibration frame. (B) Calibrating the needle tip position error in the augmented reality scenario (*navigation error*) using the augmented virtual model of the calibration frame. (C) Calibrating the needle tip position error in the augmented reality scenario locating predefined fiducial landmarks (red points) in the 3D anatomy of the spine (*navigation error with the model*).

First, a 3D‐printed calibration pad model was used, on which the pad's reference points were manually and iteratively palpated with the needle, with the operator looking directly at the pad (Figure 4). The saved coordinates of the points were used to estimate the tool tip localisation error, calculated as the root‐mean‐square (RMS) distance between the known coordinates of the reference points in the pad's CAD model and the spatial positions of the tracked needle tip when physically touching them. The localisation error obtained was 0.86 ± 0.5 mm for the configuration without the AR guide. Next, by repeating the palpation with the operator looking at the projected pad at the AR scene, a navigation error of 1.13 ± 0.3 mm was obtained. Finally, the same procedure was repeated with the spine model's landmarks, yielding an AR navigation error of 2.09 ± 0.86 mm relative to the anatomical model.

The whole estimation error protocol consisted of palpating each of the 13 fiducial points on the calibration pad 5 times, for a total of 65 punctures, as well as palpating each of the placed fiducial points 5 times, 10 at the pedicle joint of the five lumbar vertebrae and 2 at the spinal process, for a total of 60 repetitions, in a quasi‐random order.

The navigation software was implemented using a graphics engine (Unity, 2023), where the tracking of rigid objects in the physical scene is interconnected via plugins through the OTS and the RGB camera, from which image sequences are sent and projected onto virtual objects in the graphics engine.

**FIGURE 5:**
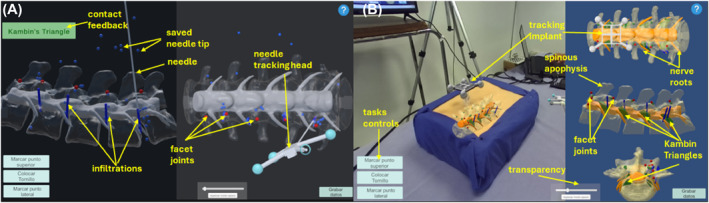
Views of the navigation simulation software with visual feedback in the form of messages and visual cues at anatomic references. (A) A simulated fluoroscopy scene with two views of the spine model in transparency. (B) A mixed reality scene with an augmented view of the patient model and orthogonal views of the virtual anatomy.

Two display modalities were implemented in the software, as illustrated in detail in Figure 5. The first was a simulated traditional fluoroscopy view (*fluoroscopy simulation*, FS), and the second was a mixed‐reality scene created by combining augmented and virtual reality views across three sections of the spine model (*mixed‐reality navigation*, MRN). In FS, lateral and anteroposterior views of the virtual lumbar spine are presented transparently, similar to the real x‐ray view, including the vertebrae from L1 to L5 and the spinal cord with its nerve roots. During the interaction, the FS shows static, frozen images of the model with the current needle projection upon verbal request from the trainees, as well as the needle tip from previous needle projections during navigation, in blue. It also represents, in blue segments, the infiltrations performed.

In MRN, the system fluidly displays a real‐time visualisation of all elements in the scene. This includes the spine model superimposed on the patient model, in the enlarged view from the RGB camera perspective, as well as the anteroposterior, lateral, and axial orthogonal views of the spine model. The spinal cord and nerve roots are shown in orange, and Kambin's triangles are represented in green. Reference points at facet joints are red, and the injection sites are shown in blue segments.

Both the FS and MR scenes include text labels that provide feedback on the contact areas, as well as buttons to control the start and end of tasks, the transparency, and the activation/deactivation of the models' anatomical structures, at the practitioner's request. This allowed for an experimental comparison of participants' performance and experiences in KTL tasks under both guiding conditions: traditional fluoroscopy and mixed‐reality navigation.

### 2.3 Experiments

#### 2.3.1 Recruitment & Study Approval

This study was carried out as a workshop for residents. It was conducted at the Center for the Improvement of Medical Skills and Abilities (CePHaDeM) at the General Hospital of Mexico ‘Dr. Eduardo Liceaga’. Ten participants were invited from residents in their second to fourth year of orthopaedic training. Participants had no prior experience with lumbar spine procedures or with KTL mannequin‐based training systems. The experiments involved comparative experiences performing KTL under 2 FS and MRN guiding conditions. The Directorate of Health Education and Training at the HGM reviewed the ethical aspects of the study and approved it as a workshop for residents.

#### 2.3.2 Assigned Tasks

Participants were assigned the task of aligning and placing the needle in four different target locations, corresponding to Kambin's Triangles in the intervertebral spaces between L1 and L5 of the spine, as it would be performed during endoscopic approaches or when targeting the intervertebral discs (Kambin Triangle Location, KTL). The targets were explicitly placed approximately at the centre of the 4 KTs in the virtual spine model.

During practice, residents received visual feedback on correct or erratic contacts at the KT and nerve roots (NR) during infiltration execution, in the form of guidelines in the orthogonal virtual planes, plus warning messages and colour cues in the spatial projection of the augmented reality view. Specifically, when correctly accessing the areas of interest in the facet joints, nerve roots, transverse process, and the corridors formed by Kambin triangles in the intervertebral regions of the vertebrae L1–L5, or when incorrect contact occurs in the nerve roots.

Participants could first practice on an open spine model without the skin (open model) for a few minutes to become familiar with the system. Subsequently, they performed the practice sessions with the spine immersed in the patient's model (closed model).

For practical purposes, to avoid modifying the assembly of the system elements within a simulated operating room at CePHaDeM, and due to the residents' time constraints, the experiments were limited to the left side of the spine. However, the system can record procedures on both sides of the patient model.

#### 2.3.3 Metrics of Performance and Precision

Quantitative data from the simulations were collected, including the time taken to complete each procedure and the accuracy of the surgical site reached. These metrics were used to evaluate the residents' performance throughout the procedure.

The accuracy of needle insertion was measured as the distance between the final tip point and the target point. To this aim, the target points were previously established manually on the 3D spine virtual model, one target point per intervertebral space from L1–L2 to L4–L5, at the corresponding KT centroid. At each infiltration, after the practitioner verbally reported having collocated the needle in the KT, an experimenter saved the final tracked tip point of the needle, and the software calculated the distance from this last position to the predefined position of the target.

After completing the tasks of targeting the 4 KTs at the intervertebral spaces, a sagittal image of the final locations of the four needle insertions was recorded at the end of the session and visually analysed by a medical expert in KTL to determine whether the tasks were correctly or incorrectly performed. A successful infiltration was considered to have occurred if the tip of the needle was observed approximately over the KT centroid and oriented approximately over the midline of the intervertebral corridor within the KT, within the lower border of the superior pedicle, and the upper border of the inferior pedicle [3].

During the tests, the simulator also recorded incidences of structural collisions with the vertebrae and nerve roots. The system recorded performance metrics, which were statistically analysed after the simulations.

#### 2.3.4 Navigation Experience

A questionnaire comprising seven items was administered after practicing both simulation methods to evaluate the practitioner's experience. The questions and their respective classifications are shown in Figure 2. A Likert scale was used to score the questions, with the following response levels and corresponding values: 1 for ‘Completely Disagree,’ 2 for ‘Disagree,’ 3 for ‘Neutral,’ 4 for ‘Agree,’ and 5 for ‘Completely Agree.’

Items in the questionnaire assessed aspects such as the perceived operability and utility of navigation for performing the tasks, and how difficult these were perceived. The complete questionnaire is presented in Table 1.

**TABLE 1:** The questionnaire was used to evaluate the practitioner's experience with the simulator.

ID Question	Question	Assessment	Term which defines
Q1	I found using the simulator easy.	Operability	Simple
Q2	The use of the tool was intuitive.	Operability	Intuitive
Q3	The movement of the tool on the screen corresponded to its movement in reality.	Operability	Consistent
Q4	The view on the screen helped me orient myself within the patient.	Utility	View
Q5	It was easy for me to spatially locate the target points with the views provided by the simulator.	Utility	Location
Q6	Visual guides (warnings and colours, if applicable) helped me perform tasks better.	Utility	Guides
Q7	Locating Kambin's triangles was simple.	Task	Kambin

#### 2.3.5 Statistical Analysis

Inferential statistical tests were applied to test the hypothesis that MRG improves training efficiency. First, after confirming the normality of the observed metrics by Kolmogorov tests, two series of multivariate analyses of the variance (MANOVA), following a 2 *Navigation Conditions* (FS, MRN) × 4 *Target Locations* (KTs between L1 and L5) design, were applied over the observed time for completing the task (*time task*) and the accuracy in placing the tip position of the needle with respect to the target points (*target error*).

The Mann‐Whitney test was applied to the self‐rated questionnaire scores, assessing participants' experience with the simulator and comparing the FS and MRN conditions.

The analysis was carried out using the SPSS v.15 software.

## 3 Results

Nine residents completed the programme, so one volunteer's metrics were discarded. The normality of the data was subsequently analysed and confirmed. Subsequently, a MANOVA parametric analysis was carried out. As shown in Table 2, the MANOVA revealed a main effect of navigation condition on task time (F(1,71) = 17.6, *p* < 0.0001), with significantly shorter execution time during MRN than during FS, and a large effect size (*η* ^2^ = 0.289). On the other hand, a slightly smaller *target error* under FS than under MRN was observed, but it was not significant, with a low effect size (*η* ^2^ = 0.013), indicating no differences in precision of needle placement between the two conditions. No main factor effect *of target location* was revealed for *target error* and *task time*, as presented in Table 3.

**TABLE 2:** Observed mean performance metrics for the location of the target points by comparing the FS versus MRN.

	Navigation condition			
	Fluoroscopy	Mixed reality	*p*‐value	*η* ^2^
Task time [s]	142 ± 109	58 ± 48	< 0.0001	0.289
Error [mm]	11.28 ± 6.25	10.1 ± 2.9	NP	0.013

**TABLE 3:** Observed performance metrics in the location of the specific target points at the Kambin triangles.

	Target location			
	L1–L2	L2–L3	L3–L4	L4–L5
Fluoroscopy simulation				
Task time [s]	114 ± 80	126 ± 56	123 ± 66	139 ± 52
Error [mm]	11.56 ± 4.64	10.41 ± 6.82	12.53 ± 7.71	10.47 ± 6.77
Mixed reality				
Task time [s]	48 ± 18	83 ± 88	49 ± 31	41 ± 21
Error [mm]	10.09 ± 3.51	9.21 ± 3.35	10.2 ± 1.91	11.01 ± 3.33

**FIGURE 6:**
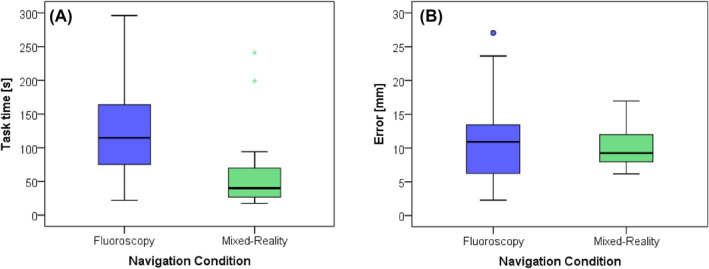
Boxplots with the median and interquartile ranges of the observed metrics of the practitioners, comparing the FS and MRN for targeting the Kambin triangles. (A) The median task time for locating the target points. (B) The median spatial error when locating the target points.

**FIGURE 7:**
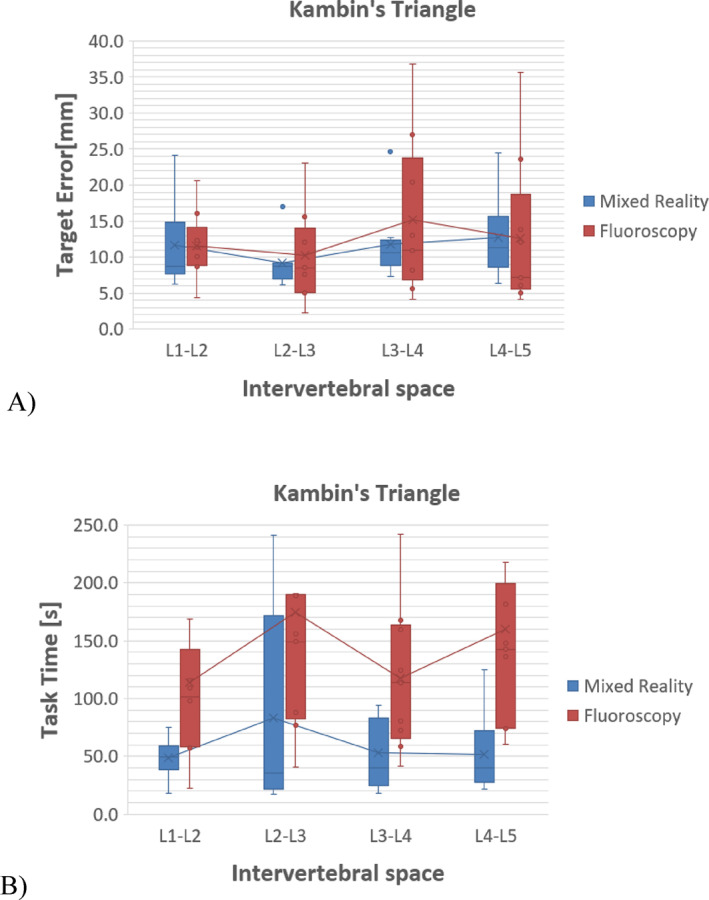
Observed metrics of median and interquartile ranges of the practitioners performing the Kambin's Triangle Localisation tasks at specific target points, comparing FS and MRN sessions. (A) Errors were achieved at KT's target for the four intervertebral spaces. (B) Time for performing the KTL at the four intervertebral spaces.

In addition, Figures 6 and 7 show the boxplots of the descriptive metrics of Tables 2 and 3, respectively, to illustrate the dispersion of the metrics across the interquartile ranges, for both FS and MR conditions, as well as for each of the specific target points in the Kambin triangles. Consequently, Figure 6 shows that in addition to minor location errors and shorter execution times, the dispersion of metrics was smaller during MRN than during FS, according to the observed interquartile ranges, indicating differences between both conditions. Figure 7 shows the distribution of the metrics along the specific target locations at KTs, also showing less dispersion among participants, according to the registered interquartile ranges at each particular target location. Interestingly, although not significant, greater error variation is observed throughout the four intervertebral spaces in needle placements by fluoroscopy simulation. Furthermore, less dispersion in errors is observed during MRN. Thus, the performance was generally better in accuracy and execution time for all the target locations under MRN.

Table 4 presents the joint probabilities of the contacts that occurred in the target zones within the Kambin triangles and the risk contacts in the nerve roots, for both FS and MRN conditions. From this, analysing the spatial localisation of the final needle placement, it was found that of the total number of trials, 70% were correctly placed in the target zones of the KT during FS, according to its marginal probability KTT for FS. In comparison, remarkably, 88% were correctly positioned with the help of MRN, considering its marginal probability KTT for MRN. On the other hand, in 5% of the punctures where some KT were contacted, contacts also occurred in the risk zones of the nerve roots, which implies that in 93% of the infiltrations during FS where the KT were reached, there was no risk contact (65% of 70% trials when the KT was contacted).

**TABLE 4:** Observed joint probability of contacts at any KT and contacts at nerve roots during infiltrations.

	Contacts					
	Fluoroscopy			Mixed reality		
	KTT	KTF	Marginal NR	KTT	KTF	Marginal NR
NRT	0.05	0	0.05	0.5	0	0.5
NRF	0.65	0.3	0.95	0.38	0.12	0.5
Marginal KT	0.7	0.3		0.88	0.12	

*Note:* KTT and KTF denote true and false contacts at any KT, while NRT and NRF denote true and false contacts at any nerve root.

In contrast, a higher incidence of contacts was observed in the KT areas of 88%, as indicated by the marginal probability of KTT, but also with a 50% increase in risk contacts during infiltrations when the KT was also contacted.

**FIGURE 8:**
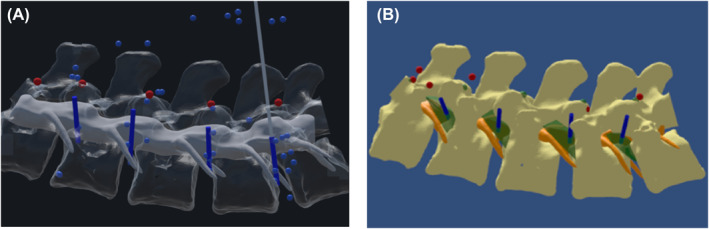
Final location of the needle within the 4 KT spaces. (A) Two incorrect placements are observed in FS. (B) Four correct placements are observed in MRN.

Figure 8 illustrates the result of the final placement of the needle in the 4 KTs for the FS and MRN sessions. The figure shows the outcomes of four infiltrations by a resident under FS and MRN. Two incorrect placements were observed in the first, while the second showed four correct placements.

**FIGURE 9:**
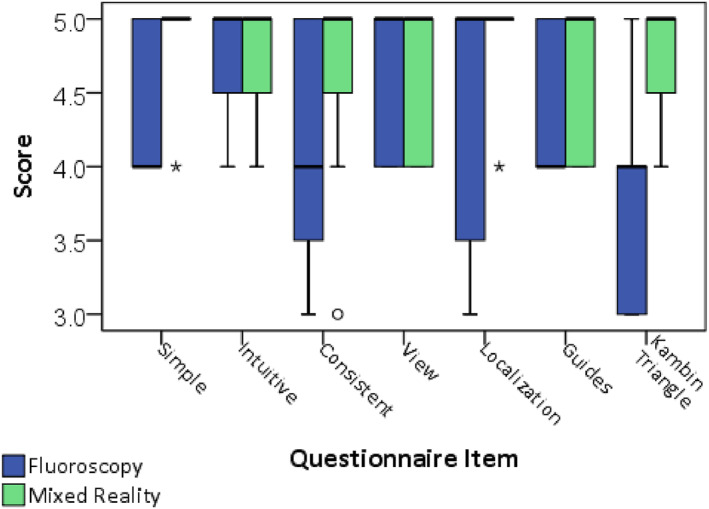
Observed scores of the questionnaire assessing perceptual aspects of operativity, utility and difficulty during the execution of KTL with the simulator, comparing the FS and MRN navigation.

Regarding the evaluation of the system by the residents, as seen in Figure 9, the five perceptual aspects assessing the perceived experience received positive median scores (≥ 4.0) from the participants in both simulated conditions. For the FS scenario, four items have a median score of 4, and three have a median score of 5. Remarkably, all seven items received the highest positive median score of 5 for the MRN scenario, despite the occurrence of some outliers. Even if the observed scores in the MRN condition presented higher values for five items than in the FS condition, no significant difference for any item was found among both simulation conditions by the non‐parametric Kruskall‐Wallis tests applied to the items' scores.

These results indicate that the navigation was well perceived as easy to operate, and useful in executing the KTL procedures of the spine. Even if not significantly different, it is worth highlighting that residents perceived the execution of KTL to be easier (*Kambin Triangle* item) under the visual aid provided by MRN.

## 4 Discussion

This work evaluated a pilot didactic plan for training spine infiltrations, combining theoretical and practical sessions with a mixed‐reality simulator. The simulator incorporates a mannequin that includes a 3D printed model of the lumbar spine from a CT study of a patient, along with navigation software that provides visual guidance combining virtual and augmented reality, in which orthogonal views of the 3D virtual model of the lumbar spine are presented, plus the same model projected and superimposed over the physical patient model in an augmented reality scene. During practice, residents received feedback in the form of successes and errors during the execution of infiltrations at target points of the spine in the form of guidelines in the orthogonal virtual planes, plus warning messages and colour cues in the spatial projection in the augmented reality view. Specifically, when correctly accessing the areas of interest in the corridors formed by the Kambin triangles in the intervertebral regions of the vertebrae (L1–L5), or when incorrect contact occurs in the nerve roots.

After a detailed theoretical session about the Kambin Triangle infiltration, a group of residents participated in practical training sessions with the simulator. The experimental study generated interesting results by comparing two experimental conditions: infiltrations with simulated fluoroscopy and mixed‐reality navigation. First, after rating the outcome of all the infiltrations with the system one by one by the expert experimenter, it was very interesting to find that the participants correctly placed the needle within the Kambin Triangle in up to 88% of the attempts. In comparison, correct placement was drastically reduced to 50% during simulated fluoroscopy guidance. In terms of execution time, participants were found to be able to complete needle placement in significantly less time—up to three times less time during the mixed‐reality navigation than during fluoroscopy guidance, on average. This makes sense when we compare the experiences reported by residents during the experiments. Looking at the differences in scores for some of the items in the questionnaire, residents generally reported having slightly greater ease in understanding the anatomy and the clinical case during the mixed‐reality guidance and having perceived locating target points more easily under this condition. This is explained, among other aspects, by the fact that during mixed reality, the system allows the needle to be followed in real‐time and its location to be viewed continuously with respect to the model in three dimensions, superimposed on the 3D printed model of the lumbar spine. On the contrary, during fluoroscopy, residents received feedback on the position and orientation of the needle only for instants, simulating fluoroscopy by radiating the patient for moments discontinuously. Furthermore, the same fluidity of the system's information during augmented reality allows the resident to be provided with visual information in the form of colours and warning messages instantly, which was reflected in the slightly higher opinion on the usefulness of augmented reality in executing infiltrations safely.

Regarding safety in the simulated procedures, after analysing the contacts made in the area of interest at the KTs and the risk areas in the nerve roots, it was found that during the mixed reality‐guided training, the residents made more risky contacts in the nerve roots than during the fluoroscopy‐guided infiltrations, even though the occurrence of correct placements was higher during the mixed reality‐guided condition. In fact, 93% of the infiltrations in which the Kambin Triangle was reached by fluoroscopy were safe, that is, without any contact recorded in the nerve roots, representing 65% of the total infiltrations. The opposite case, which is noteworthy, occurred with the infiltrations guided by mixed reality, where despite showing a high success in the final placement, these were achieved at the cost of making unsafe contacts in the nerve roots 63% of the times that the Kambin Triangle was reached, presenting this event in 50% of the total the trials, being successful or unsuccessful infiltrations. This high rate of nerve contact might be interpreted in the context of the study population: orthopaedic residents with no prior experience in this specific intervention. Consequently, a portion of this risk may be attributed to the learning curve inherent to this user group. This also may indicate that although mixed reality was of great benefit for spatial coordination and location, as well as understanding and locating the anatomical areas, the movements being fluid, the system did not have sufficient warning mechanisms for movement restrictions that would allow residents to be warned more adequately of the risk during their manoeuvres. In contrast, with fluoroscopy, since needle placement and orientation are achieved in phased movements, the procedures require a greater attention load, more restricted movements, and therefore more prolonged procedures.

Another explanation may be that due to the feeling of ease of interaction, fluidity, and visuospatial understanding with augmented reality, of movements and anatomy, they acquired a greater level of confidence and security with the visual guidance provided by mixed‐reality and, consequently, influenced them to inadvertently feel motivated to execute more risky gestures and manoeuvres, which they possibly would have avoided during fluoroscopy, or that would not have been easy to execute.

Furthermore, analysing the performance of the trainees by comparing the two simulation conditions, we found no differences in terms of the observed accuracy in the final placement of the needle tip concerning the target point within the Kambien Triangles, with a maximum error, discounting an outlier, of up to about 25 mm during fluoroscopy simulation, but about 17 mm maximum during mixed‐reality. Additionally, a greater dispersion in positioning errors was observed during fluoroscopy than in mixed reality. These errors suggest an advantage in reaching target points with the help of mixed reality, but they still need to be confirmed with further experiments.

In any case, it would be expected that mixed reality allows for a significant reduction in errors. Therefore, based on the observed safety profile, we must acknowledge that although the technique shows considerable potential, it is not yet suitable for routine clinical use. Instead, currently, it serves as a high‐fidelity educational tool for training. Before clinical translation, further development is warranted to reduce the system's tracking error and improve reliability. More research is needed to better adapt representative visual information during mixed reality, such as different viewpoints, virtual planes, linear guidelines, and tolerances, among other interactive cues and constraints, to consistently better warn of risks and anticipate contacts before they occur in sensitive areas.

Finally, the experiences reported by the residents indicate that the simulator was well‐evaluated. They found it helpful to understand the anatomy and the clinical case operated on, even to the point of having the sensation of infiltrating a real patient. It can be argued that the simulator is suitable for educational strategies, combining theoretical and practical sessions. Future work also includes conducting longitudinal efficacy validations during a multi‐session educational programme and corroborating successful outcomes of the infiltrations in the model using radiological computed tomography scans.

## 5 Conclusion

In this study, we present an evaluation of a new training simulator for Kambin Triangle infiltration procedures of the spine that combines virtual fluoroscopy and mixed‐reality navigation.

Residents reported greater ease in understanding the tasks to be performed and the anatomy of the patient's clinical case under mixed‐reality practice. The experiments demonstrated that residents showed a dramatically higher number of successful infiltrations during mixed‐reality navigation. However, they also showed higher‐risk nerve root contacts during this navigation than during simulated fluoroscopy. This suggests, on the one hand, the potential advantages of first practicing under mixed‐reality guidance to more effectively understand the anatomical aspects of the approach, followed by practice with simulated fluoroscopy, resulting in training that is increasingly closer to reality. However, on the other hand, it emphasises the need to incorporate better visual guidance mechanisms and enhanced augmented reality views to more effectively warn residents of potential errors and risky manoeuvres during simulation before their first contact with real patients.

In any case, the observed results and the verbal opinions of the residents and experts indicate that the system is valuable and suitable for educational and training purposes. However, regarding clinical applicability, the current incidence of nerve root contact indicates that the system is not yet suitable for routine intraoperative use. Further development regarding tracking accuracy and safety warnings, design of educative training programs and protocols, and combining sessions of mixed‐reality and fluoroscopy scenarios. Additionally, it provides preliminary evidence on the potential benefits of adopting mixed‐reality navigation schemes in the future in clinical practice.

## Author Contributions

Conceptualization: C.F.D.‐V., J.A.‐F., F.C., V.F., and M.A.P.‐C. Methodology: J.A.‐F., J.A.P.‐T., F.M.C.‐J., M.A.C.‐G., and M.A.P.‐C. Software: C.F.D.‐V., J.A.‐F., and F.C. Validation: J.A.‐F., J.A.P.‐T., and M.A.P.‐C. Formal analysis: C.F.D.‐V., J.A.P.T., E.A.‐R., and M.A.P.‐C. Investigation: C.F.D.‐V., E.A.‐R., and M.A.P.‐C. Resources: M.A.P.‐C. Data curation: J.A.‐F., and M.A.P.‐C. Writing original draft preparation: C.F.D.‐V., E.A.‐R., V.F., and M.A.P.‐C. Writing – review and editing: C.F.D.‐V., E.A.‐R., V.F., and M.A.P.‐C. Supervision: F.M.C.‐J., M.A.C.‐G., and M.A.P.‐C. Project administration: M.A.P.‐C. Funding acquisition: M.A.P.‐C. All authors have read and agreed to the published version of the manuscript.

## Funding

This research was funded by DGAPA UNAM, Mexico, (Grant DGAPA‐PAPIIT IN117425), and Secretaria de Educación, Ciencia, Tecnología e Innovación de la Cd. México SECTEI, Mexico, (Grant SECTEI 087/2023).

## Ethics Statement

The study was approved by the Directorate of Health Education and Training of the General Hospital of Mexico ‘Dr. Eduardo Liceaga’ as a training workshop for residents.

## Consent

The authors have nothing to report.

## Conflicts of Interest

The authors declare no conflicts of interest.

## Data Availability

Research data are shared upon on reasonable request.

## References

[1] M. G. Baabor , M. R. Silvas , and E. D. M. Martínez , “Transforaminal Percutaneous Approach: Kambin's Safety Triangle,” NeuroTarget 12, no. 2 (2018): 6–9, 10.47924/neurotarget2018105.

[2] T. Li , G. Wu , Y. Dong , Z.‐Q. Song , and H. Li , “Kambin's Triangle‐Related Data Based on Magnetic Resonance Neurography and Its Role in Percutaneous Transforaminal Endoscopic Lumbar Interbody Fusion,” Journal of Orthopaedic Surgery and Research 17, no. 1 (2022): 543, 10.1186/s13018-022-03428-3. 36522770 PMC9756519

[3] N. Nikpour , Z. Fazelinejad , M. Sametzadeh , M. A. Lordjani , and A. R. E. Moghadam , “Anatomical Assessment of the Kambin's Triangle for Percutaneous Posterolateral Transforaminal Endoscopic Surgery of Lumbar Intervertebral Discs: A Magnetic Resonance Imaging Based Study,” Anatomy & Cell Biology 57, no. 4 (2024): 523–534, 10.5115/acb.24.112. 39511780 PMC11663528

[4] S. E. Glaser and R. V. Shah , “Root Cause Analysis of Paraplegia Following Transforaminal Epidural Steroid Injections: The ‘Unsafe’ Triangle,” Pain Physician 237 (2010), 10.36076/PPJ.2010/13/237. 20495587

[5] K. Trinh , C. Gharibo , S. M. Aydin , and S. M. Aydin , “Inadvertent Intradiscal Injection With TFESI Utilizing Kambin's Retrodiscal Approach in the Treatment of Acute Lumbar Radiculopathy,” Pain Practice 16, no. 4 (2016), 10.1111/PAPR.12420. 26896050

[6] A. A. Fanous , L. M. Tumialán , and M. Wang , “Kambin's Triangle: Definition and New Classification Schema,” Journal of Neurosurgery (2020), 10.3171/2019.8.SPINE181475. 31783346

[7] T. Q. Tabarestani , D. A. W. Sykes , G. Maquoit , et al., “Novel Merging of CT and MRI to Allow for Safe Navigation Into Kambin's Triangle for Percutaneous Lumbar Interbody Fusion—Initial Case Series Investigating Safety and Efficacy,” Operative Neurosurgery 24, no. 3 (2023): 331–340, 10.1227/ons.0000000000000531. 36701664

[8] M. I. Yusof , A. A. Salim , J. Johari , and A. R. Rajagopal , “Determination of the Entry Point for Lower Lumbar Intradiscal Procedure Using Transforaminal Technique: Cross‐Sectional Study Using Magnetic Resonance Imaging,” Spine Surgery and Related Research 6, no. 6 (2022): 689–695, 10.22603/ssrr.2021-0129. 36561161 PMC9747223

[9] L. Manchikanti , A. D. Kaye , A. Soin , et al., “Comprehensive Evidence‐Based Guidelines for Facet Joint Interventions in the Management of Chronic Spinal Pain: American Society of Interventional Pain Physicians (ASIPP) Guidelines Facet Joint Interventions 2020 Guidelines,” Pain Physician (2020). 32503359

[10] P. Freyhardt , T. Hartwig , M. de Bucourt , et al., “MR‐Guided Facet Joint Injection Therapy Using an Open 1.0‐T MRI System: An Outcome Study,” European Radiology (2013), 10.1007/S00330-013-2940-9. 23812244

[11] T. Stauffer , Q. Lohmeyer , S. Melamed , et al., “Evaluation of Augmented Reality Training for a Navigation Device Used for CT‐Guided Needle Placement,” International Journal of Computer Assisted Radiology and Surgery 19, no. 12 (2024): 2411–2419, 10.1007/s11548-024-03112-3. 38717736 PMC11607048

[12] A. Evans , S. Shevlin , D. Burckett‐St.Laurent , J. Bowness , and A. Macfarlane , “Pilot Study Exploring if an Augmented Reality Needletrainer Device Improves Novice Performance of a Simulated Central Venous Catheter Insertion on a Phantom,” Cureus 15 (2023), 10.7759/cureus.40197. PMC10329877 37431346

[13] C. F. Domínguez‐Velasco , I. E. Tello‐Mata , G. Guinto‐Nishimura , et al., “Augmented Reality Simulation as Training Model of Ventricular Puncture: Evidence in the Improvement of the Quality of Punctures,” International Journal of Medical Robotics and Computer Assisted Surgery 19, no. 5 (2023): e2529, 10.1002/rcs.2529. 37272193

[14] T. Borde , L. Saccenti , M. Li , et al., “Smart Goggles Augmented Reality CT–US Fusion Compared to Conventional Fusion Navigation for Percutaneous Needle Insertion,” International Journal of Computer Assisted Radiology and Surgery 20, no. 1 (2024): 107–115, 10.1007/s11548-024-03148-5. 38814530 PMC11758159

[15] A. B. G. Dessai , S. K. Shetty , D. N. Jijo , and O. U. Gaonkar , “Augmented Reality System Guidance for Computed Tomography‐Based Needle Insertion: A Narrative Review,” Journal of Clinical and Diagnostic Research (2024), 10.7860/jcdr/2024/70665.19631.

[16] A. Berger , O. J. Choudhry , and D. Kondziolka , “Augmented Reality–Assisted Percutaneous Rhizotomy for Trigeminal Neuralgia,” Operative Neurosurgery 24, no. 6 (2023): 665–669, 10.1227/ons.0000000000000661. 36815787

[17] W. Qin , S. Wang , X. Chen , Y. Zhuang , Y. Shen , and Y. Shen , “Visualization System for Closed Thoracic Drainage Puncture Based on Augmented Reality and Ultrafine Diameter Camera,” Journal of Shanghai Jiaotong University 30, no. 3 (2025): 417–424, 10.1007/s12204-025-2808-6.

[18] C. A. Agten , C. Dennler , A. B. Rosskopf , L. Jaberg , C. W. A. Pfirrmann , and M. Farshad , “Augmented Reality–Guided Lumbar Facet Joint Injections,” Investigative Radiology 53, no. 8 (2018): 495–498, 10.1097/RLI.0000000000000478. 29742535

[19] S. Condino , G. Turini , P. D. Parchi , et al., “How to Build a Patient‐Specific Hybrid Simulator for Orthopaedic Open Surgery: Benefits and Limits of Mixed‐Reality Using the Microsoft Hololens,” Journal of Healthcare Engineering 2018, no. 1 (2018): 5435097, 10.1155/2018/5435097. 30515284 PMC6236521

[20] C.‐C. Chang , C.‐H. Kuo , H.‐K. Chang , et al., “Augmented Reality‐Assisted Percutaneous Pedicle Screw Instrumentation: A Cadaveric Feasibility and Accuracy Study,” Applied Sciences 12, no. 10 (2022): 5261, 10.3390/app12105261.

[21] Y. Ma , J. Wu , Y. Dong , H. Tang , and X. Ma , “Augmented Reality Navigation System Enhances the Accuracy of Spinal Surgery Pedicle Screw Placement: A Randomized, Multicenter, Parallel‐Controlled Clinical Trial,” Orthopaedic Surgery 17, no. 2 (2025): 631–643, 10.1111/os.14295. 39815419 PMC11787979

[22] A. Bardeesi , T. Q. Tabarestani , S. M. Bergin , et al., “Using Augmented Reality Technology to Optimize Transfacet Lumbar Interbody Fusion: A Case Report,” Journal of Clinical Medicine 13, no. 5 (2024): 1513, 10.3390/jcm13051513. 38592365 PMC10934424

[23] A. Fedorov , R. Beichel , J. Kalpathy‐Cramer , et al., “3D Slicer as an Image Computing Platform for the Quantitative Imaging Network,” Magnetic Resonance Imaging 30, no. 9 (2012): 1323–1341, 10.1016/j.mri.2012.05.001. 22770690 PMC3466397

[24] Blender Online Community , Blender—A 3D Modelling and Rendering Package [Software], (Blender Foundation, 2025), https://www.blender.org.

